# Combinational Antibacterial Activity of Nisin and 3-Phenyllactic Acid and Their Co-production by Engineered *Lactococcus lactis*

**DOI:** 10.3389/fbioe.2021.612105

**Published:** 2021-02-05

**Authors:** Jiaheng Liu, Rongrong Huang, Qianqian Song, Hui Xiong, Juan Ma, Rui Xia, Jianjun Qiao

**Affiliations:** ^1^Department of Pharmaceutical Engineering, School of Chemical Engineering and Technology, Tianjin University, Tianjin, China; ^2^Key Laboratory of Systems Bioengineering, Ministry of Education, Tianjin, China; ^3^Collaborative Innovation Center of Chemical Science and Engineering, SynBio Research Platform, Tianjin, China

**Keywords:** *Lactococcus lactis*, nisin, 3-phenyllactic acid, antimicrobial activity, food preservative, metabolic engineering

## Abstract

Nisin produced by certain *Lactococcus lactis* strains is commercially used in meat and dairy industries because of its effective antibacterial activity and food safety characteristics. It has been proved that the antibacterial activity could be enhanced when combined with other antimicrobial agents. In this study, we demonstrated that nisin and 3-phenyllactic acid (PLA) in combination displayed excellent combinational antibacterial activity against foodborne pathogens including *S. xylosus* and *M. luteus*. The potential application in food preservation was further verified *via* microbial analysis during the storage of meat and milk, and determination of strawberry rot rate. Scanning electron microscopy observation indicated a distinct mode of PLA with nisin, which may target at the dividing cell, contributing to their combinational antibacterial effect of nisin and PLA. Considering the positive results, a nisin-PLA co-producing strain was constructed based on the food-grade strain *L. lactis* F44, a nisin Z producer. By the knockout of two L-lactate dehydrogenase (LDH) and overexpression of D-LDH^*Y*25A^, the yield of PLA was significantly increased 1.77-fold in comparison with the wild type. Anti-bacterial assays demonstrated that the fermentation product of the recombinant strain performed highly effective antibacterial activity. These results provided a promising prospect for the nisin-PLA co-expressing *L. lactis* in food preservation on account of its considerable antibacterial activity and cost-effective performance.

## Introduction

Food safety is generally recognized as an essential public safety issue worldwide. Microbial contamination of food products is of great importance effecting food safety, which can bring about great economic losses and prevalent foodborne diseases (Chatterjee and Abraham, 2017). Although various synthetic preservatives, such as sodium benzoate, potassium sorbate, and butylated hydroxyanisole, have been investigated and applied to inhibit microbial growth during food storage, the increasingly serious threat of synthetic preservatives to human health has prompted investigations into exploring natural preservatives (KBauera et al., 2001; Przybylski et al., 2016; de Oliveira Pateis et al., 2018).

Nisin, produced by certain strains of *Lactococcus lactis*, is a natural antimicrobial peptide exhibiting broad-spectrum antimicrobial activity against a majority of Gram-positive foodborne bacteria and some Gram-negative pathogens when combined with EDTA or physical treatments (Gharsallaoui et al., 2015). It exerts its antimicrobial activity by both pore formation on the surface of cells and inhibition of cell wall biosynthesis (Chatterjee et al., 2005). More importantly, nisin is easily degradable by proteolytic enzymes in mammalian and presents no risk to human health. Thus, the application of nisin in preservation of many food products, such as cheese and meat, has a long and impressive history (Cotter et al., 2005; Barbosa et al., 2017). However, it has less significance for fruit or bread preservation due to its weak antifungal activity.

Another major problem is that pure product of nisin (>99%) is commercially unavailable due to its time-consuming separation and purification (Juturu and Wu, 2018). The commercial nisin preparation called Nisaplin contains only 2.5% (w/w) nisin, and the rest are mainly NaCl and proteins. In some cases, the nisin fermentation broth is immediately applied in food preservation to reduce the cost, while the bounding effect between nisin and proteins could decrease its preservative effectiveness (Cleveland et al., 2002).

The above issues prompted us to turn to the strategy of combined application of nisin and other antimicrobial agents. PLA, an organic acid, has been reported to be an antimicrobial compound with broad-spectrum activity against bacteria including *Listeria monocytogenes* (Dieuleveux et al., 1998), *Staphylococcus aureus*, and *Escherichia coli* (Ohhira et al., 2010), and fungi including yeasts (Schwenninger et al., 2008) and a wide range of molds, such as *Aspergillus ochraceus*, *Penicillium roqueforti*, and *Penicillium citrinu* (Lavermicocca et al., 2003b). Its antimicrobial mechanism of action is still under investigation, and some researchers supposed that the interaction between PLA and cell surface could contribute to the damage of cellular structures (Ning et al., 2017; Sorrentino et al., 2018). It has been reported that PLA could be produced by a wide range of lactic acid bacteria species, such as *Lactobacillus* and *Enterococcus* (Lavermicocca et al., 2000; Valerio et al., 2004).

Therefore, the objectives of this research were to: (1) examine whether there is a combinational antibacterial activity of nisin and PLA against foodborne pathogens, (2) evaluate the efficacy of combined application of nisin-PLA in food preservation, and (3) construct an *L. lactis* strain co-producing nisin and PLA, the fermentation product of which could exhibit both antibacterial and antifungal activities and serve as a potential preservative candidate in food industry more widely.

## Materials and Methods

### Strains, Plasmids, and Growth Conditions

All bacteria strains and plasmids used in this study are listed in Supplementary Table S1. *Lactococcus lactis* F44 (Genome accession number: PRJNA419050), a nisin Z producer, was derived from *L. lactis* YF11 *via* genome shuffling in our previous study (Zhang et al., 2014b). *L. lactis* F44 and the engineered strains were incubated at 30°C with no agitation in seed medium or fermentation medium. The seed medium contained the following (wt/vol): yeast extract (1.5%), peptone (1.5%), KH_2_PO_4_ (2.0%), sucrose (2.0%), NaCl (0.15%), and MgSO_4_⋅7H_2_O (0.015%), pH 7.2. For the fermentation medium, additional corn steep liquor (0.3%) and cysteine (0.26%) were needed. Three g/L phenylpyruvic acid (PPA) was supplemented in the fermentation medium for PLA production.

*Escherichia coli* TG1 was applied for the construction of expression vector. *Staphylococcus xylosus* and *Micrococcus luteus* ATC10240 were used as indicators and cultured in Nutrient Broth (NB). 5 μg/mL erythromycin (Em) and 5 μg/mL chloramphenicol (Cm) were employed for *L. lactis*, while for *E. coli*, 200 μg/mL Em and 50 μg/mL Cm were applied, if necessary.

### Anti-bacterial Activity

Agar diffusion assay was performed to detect the anti-bacterial activities of PLA and nisin, or the culture supernatants. Briefly, 25 mL of NB agar and 200 μL indicator strain (5 × 10^8^ CFU/mL) were mixed and poured onto a plate with Oxford cups (6 mm). The wells of the dried agar plates were added with 100 μl of samples and the plates were incubated overnight at 37°C. The standards PLA and nisin were applied to verify the combinational effect of PLA and nisin in antibacterial. The concentrations of the standards were set as follows: 10 mg/mL PLA (Liu et al., 2018), 100 IU/mL nisin (Wu et al., 2020) and a mixture of PLA-nisin (10 mg/mL PLA and 100 IU/mL nisin), and 0.02 M HCl was taken as blank. Besides, the boiled supernatant (100°C, 5 min) from fermentation sample were used to evaluate the antibacterial activities of F44/P, F44/DLDH and F44ΔLDHΔLDHB/DLDH. For the inhibitory effect against *M. luteus*, a dilution of 1:8 was used, and the culture supernatant without dilution for *S. xylosus*.

### Microbiology Analysis in Pork Samples

The microbial analysis was performed to assess the application of PLA and nisin in pork preservation with a modified method (Liu et al., 2020b). Fresh ground pork was purchased in local market (Tianjin, China). It was divided into 4 groups and mixed with sterilized water in a ratio of 1 g/1 mL. Three experimental groups were supplemented with nisin and PLA in a final concentration of 0.1% (wt/vol), and 0.1% nisin-0.1%PLA mixture in combination, respectively. Sterilized water served as the blank. Subsequently, the samples were stored in sterile containers at 4°C. 5 g sample was taken at days 1, 2, and 3 and mixed with 30 mL saline to calculate the number of microorganism. One milliliter supernatant was serially diluted with saline and 100 μL of dilution was spread on the NB agar plate. The plate was incubated at 30°C for 24 h. The results were expressed as log_10_ CFU per gram of pork (log_10_ CFU/g). All the samples were carried out in triplicate.

### Microbiology Analysis in Pasteurized Milk

*S. xylosus* belongs to Staphylococcus, one of the microbiota in many dairy products and meat products, and has strong drug resistance (Martuscelli et al., 2000). *S. xylosus* (10^3^ CFU/mL) was added to 1 L of pasteurized milk and divided into 4 groups which were mixed with 0.1% nisin solution, 0.1% PLA solution, and 0.1% nisin-0.1% PLA solution, respectively. The mixtures were stored in sterile containers at 4°C. 1 mL of pasteurized milk sample was carried out at 0, 3, 6, and 9 h to count the number of *S. xylosus via* spreading an appropriate dilution on the NB agar plate. The result was showed as log_1__0_CFU per milliliter pasteurized milk (log_10_ CFU/mL). All the samples were tested in triplicate.

### Determination of Rotting Rate in Strawberry

Strawberries with uniform size and no visible damage were selected in a local farm. The rotting rate was determined with a modified method (Liu et al., 2020b). Then 100 strawberries were randomly divided into 4 groups, dipping in sterilized water as blank and the solution of nisin, PLA, and nisin-PLA mixture for 30 s, respectively, and the concentration of each preservative was 0.1% (wt/vol). The strawberries were stored at room temperature after air drying in Clean Bench. The number of rotten strawberries with mildew, injury, or black spot surface, was counted at days 0, 1, 2, 3, 4, 5, and 6. The rotting rate was calculated as the following:

$$R\,o\,t\,t\,i\,n\,g\,r\,a\,t\,e=\frac{t\,h\,e\,n\,u\,m\,b\,e\,r\,o\,f\,r\,o\,t\,t\,e\,n\,s\,t\,r\,a\,w\,b\,e\,r\,r\,i\,e\,s}{t\,h\,e\,i\,n\,i\,t\,i\,a\,l\,n\,u\,m\,b\,e\,r\,o\,f\,s\,t\,r\,a\,w\,b\,e\,r\,r\,i\,e\,s}\times 100\%.$$

### Scanning Electron Microscopy (SEM)

*S. xylosus*, grown to logarithmic phase, was collected and washed twice with PBS buffer. The cells were treated with nisin (50 IU/mL), PLA (5 mg/mL) or their combination and 0.9% NaCl solution (control) for 1 h at 37°C. After incubation, the cells were collected, washed with PBS and fixed in 2.5% glutaraldehyde overnight at 4°C. Following washes of three times with the same buffer, the samples were dehydrated with ethanol of gradient concentrations (50, 70, 80, 90, 100%). The samples were lyophilized and prepared for SEM (Hitachi High-Technologies, Tokyo, Japan) analysis.

### Construction of Plasmids and PLA Producing Strains

Primers used in this study were listed in Supplementary Table S2. *d-ldh^*Y*52A^* from *Lactobacillus pentosus* (Ishikura et al., 2005), encoding lactate dehydrogenase mutant D-LDH^*Y*25A^, was synthesized in GENEWIZ Inc. (Suzhou, China). Seamless cloning technology was applied to construct the *d-ldh^*Y*52A^* expression vector. The expression fragment *d-ldh^*Y*52A^* was amplified using Phanta Max Super-Fidelity DNA polymerase (Vazyme Biotech Co., Ltd., Nanjing, China) and purified with Universal DNA Purification kit (TIANGEN, Biotech, Beijing, China). Then, the fragment was cloned into *Hin*dIII-*Bam*HI digested pLEB124 to produce pLEB124-DLDH *via* Minerva Super Fusion Cloning Kit (US EVERBRIGHT^®^ INC.). The recombinant plasmid was transformed into *E. coli* TG1 and then electroporated into *L. lactis* F44 and F44ΔLDHΔLDHB, respectively, obtaining the overexpressing strains F44/DLDH and F44ΔLDHΔLDHB/DLDH.

For knockout of lactate dehydrogenase genes *ldh* and *ldhB* in *L. lactis* F44, an *oroP*-based selection/counterselection vector pCS1966 was employed (Solem et al., 2008). The upstream and downstream (∼1,000 bp) of *ldh* or *ldhB* were inserted into the plasmid pCS1966 to constructed the recombinant plasmid pCS1966-LDH or pCS1966-LDHB. The plasmid pCS1966-LDH was then introduced into F44 and the successful integration was selected by Em resistance. Subsequently, the transformant was plated on SA glucose plates supplemented with 5-fluoroorotate (5-FOA, 20 μg/mL) to screen the resistant strain where the secondary homologous recombination was occurred. Eventually, the *ldh-*deleted strain F44ΔLDH was gained. In the same method, the double knockout strain F44ΔLDHΔLDHB was obtained.

### Determination of PLA and PPA Contents in Fermentation Broth

The 12 h-culture supernatants of F44/P, F44/DLDH and F44ΔLDHΔLDHB/DLDH were collected for content analysis of PLA and PPA by high performance liquid chromatography (HPLC), equipped with a HC-C18 column (3.5 μm, 4.6 × 250 mm, Agilent) and a 2489 UV/Vis Detector. A gradient separation was programmed with the mobile phase of 0.05% trifluoroacetic acid (TFA)-0.05% TFA methanol and at a flow rate of 1 mL/min. The detection wavelength was 210 nm. The PLA and PPA standards were purchased from Meryer (Shanghai, China).

### Statistical Analysis

Experimental data in this study were expressed as means ± standard deviations (SD). SPSS 18.0 software (SPSS, Chicago, IL, United States) was used for the statistical analyses. One-way analysis of variance (ANOVA) was applied to determine the differences for diameter of inhibition zone, microbial count, rotting rate, and PLA concentration between experimental and control groups. A *p* < 0.05 was considered statistically significant.

## Results

### Combinational Antibacterial Activity of Nisin and PLA

**FIGURE 1:**
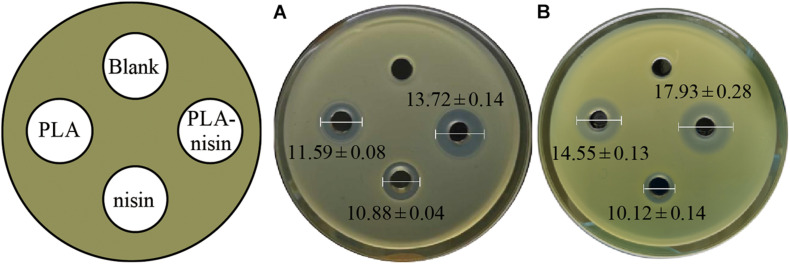
The antibacterial activities of PLA and nisin against *S. xylosus* **(A)** and *M. luteus* **(B)**. The wells (6 mm) of the plates were added with 100 μL 0.02 M HCl as blank, 10 mg/mL PLA, 100 IU/mL nisin and a mixture of PLA-nisin with the same concentrations. The differences in inhibition diameters (unit: mm) represents bacteriostasis activities of PLA and nisin. The differences among the four groups were compared by One-way ANOVA. The diameter of inhibition zone of PLA-nisin was significantly different (*P* < 0.01) compared with PLA, nisin and control.

To verify whether the combination of nisin and PLA possessed a positive effect in antimicrobial activity, pathogens of *M. luteus* and *S. xylosus* were used as indictors. The suppressive effects of PLA (10 mg/mL), nisin (100 IU/mL), nisin-PLA (mixture of 10 mg/mL PLA and 100 IU/mL nisin) were compared, with 0.02 M HCl served as negative control, and the result was shown in Figure 1. Both PLA and nisin showed antibacterial activities against *M. luteus* and *S. xylosus*. The control group, by contrast, exhibited no inhibition zone. Larger diameters of inhibition zones of nisin-PLA (13.72 ± 0.14 mm for *S. xylosus* and 17.93 ± 0.28 mm for *M. luteus*) were observed, than those of PLA and nisin alone, suggesting a superior antibacterial activity of the combined nisin-PLA. Interestingly, PLA appeared to work better in inhibiting the growth of *M. luteus*, while for nisin, slight difference was observed between the two indicators. Similarily, the combined nisin-PLA also performed a stronger bacteriostatic efficacy against *M. luteus*. All these results demonstrated that the antibacterial efficacy of nisin was substantially enhanced when combined with PLA.

### Preservation Effect of Nisin and PLA on Fresh Pork

**FIGURE 2:**
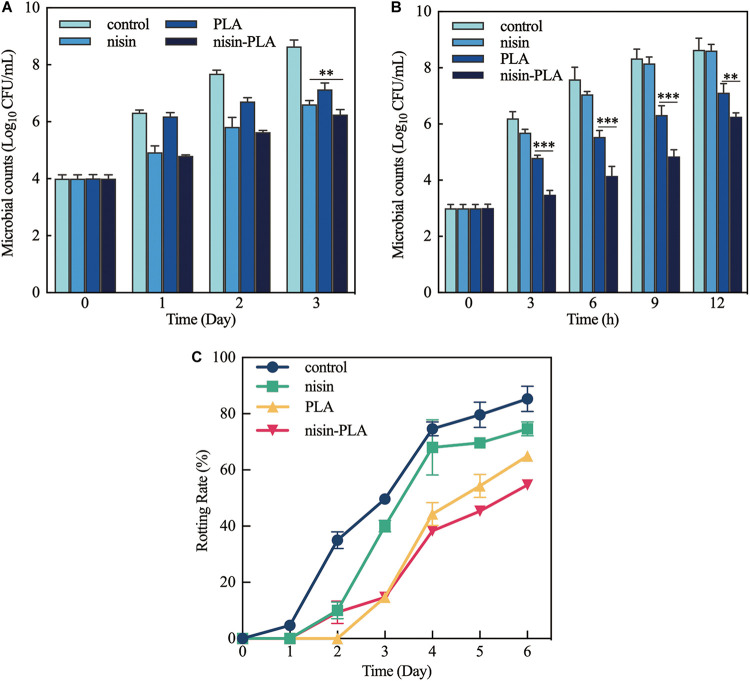
Effects of food preservation of nisin and PLA alone, or in combination. **(A)** Microbial changes in chilled pork stored at refrigerator (4°C), **(B)** microbial changes of pasteurized milk at a starting inoculum concentration of 10^3^ CFU/mL, and **(C)** the rot rate of strawberry during storage at room temperature. The samples were treated with sterilized water (control), nisin (0.1%), PLA (0.1%), and nisin (0.1%)-PLA (0.1%), respectively. The data were carried out in triplicate and the standard deviation (SD) of data was shown as error bar. The differences between the four groups were determined by One-way ANOVA, and the significantly differences (**P < 0.05; ***P < 0.01) between the groups were presented in the figure.

To evaluate the application of a combination of nisin and PLA in food preservation, chilled pork was taken as an example. Considering microorganisms are the major cause of food spoilage, microbial colony counting was used to assess the spoilage degree of chilled pork. As shown in Figure 2A, the growth of microbials in which groups were mixed with 0.1% nisin and 0.1% PLA alone or in combination, was effectively suppressed, compared with that of the sample treated with sterilized water. The viable count of microbials was reduced by more than 2 log_10_ CFU/mL in the group treated with the combination of nisin and PLA at day 3. However, the microbial count in the combination one (6.25 ± 0.27 log_10_ CFU/mL) was slightly better (*p* < 0.05) than that of the nisin-treated one (6.61 ± 0.35 log_10_ CFU/mL) at day 3, indicating a dominated role of nisin in pork preservation, instead of PLA.

### Preservation Effect of Nisin and PLA on Pasteurized Milk

To further investigate whether nisin and PLA in combination had the advantages of inhibiting the growth of bacteria in food preservation, the antibacterial activity in pasteurized milk was evaluated. As fresh milk is usually kept in the refrigerator after opening, the experiment was carried out at 4°C. One box of Pasteurized milk after incubation of *S. xylosus* was divided into four parts and mixed with sterilized water, 0.1% nisin and 0.1% PLA alone or in combination, respectively. Figure 2B showed that the addition of PLA alone or nisin and PLA in combination significantly slowed down the growth of *S. xylosus*, and the inhibition effect was especially remarkable when the storage time was less than 9 h. However, the inhibition effect of nisin was weak and no obvious difference in viable microbial counts was observed between the nisin-addition sample and the control sample. The viable microbial count of the sample treated with nisin-PLA was decreased almost 3.5 log_10_ CFU/mL before 9 h and the number of microbials was lowest, which decreased 27.5% (*p* < 0.01), 25.2% (*p* < 0.01), 23.5% (*p* < 0.01), and 12.1% (*p* < 0.05) compared with those of the PLA-treated group, respectively, at 3, 6, 9, and 12 h. Interestingly, unlike the preservation effect in fresh pork which nisin played the chief role, PLA performed better antibacterial activity in this assay. These results further illustrated that a combination of nisin and PLA exhibited an enhanced effect in Pasteurized milk preservation against *S. xylosus*.

### Preservation Effect of Nisin and PLA on Strawberry

The increasing demand for fruit products, as well as maintaining their natural characteristics (Olaimat and Holley, 2012), makes nisin as a candidate in the preservation of fruits and vegetables in recent years (Komitopoulou et al., 2001; Barbosa et al., 2017). We speculated whether nisin in the presence of PLA, could have a better performance on fruit preservation. Strawberry, easy to be damaged and rotten, was chosen for the test. The fresh strawberries were dipped in sterilized water, 0.1% nisin and 0.1% PLA alone or in combination for 30 s, respectively, kept dry and stored at room temperature. The rotting rates were summarized in Figure 2C. The rotting rates of three experimental groups were lower than those of the control in 6 days, especially the groups treated with 0.1% PLA alone and nisin-PLA in combination, which was corresponding with the results presented in the Pasteurized milk preservation assay. The rotting rate in the PLA-treating group performed better within 3 days, while the combined nisin-PLA treatment was more effective (*p* < 0.05) in days 5 and 6 which the rotting rates dropped considerably (35% decrease in day 5 and 30% decrease in the day 6) than those of the control. Significantly, the combination of nisin and PLA demonstrated better potential in the fruits preservation in comparision with nisin.

### Effect of Nisin and PLA on Bacterial Morphology

**FIGURE 3:**
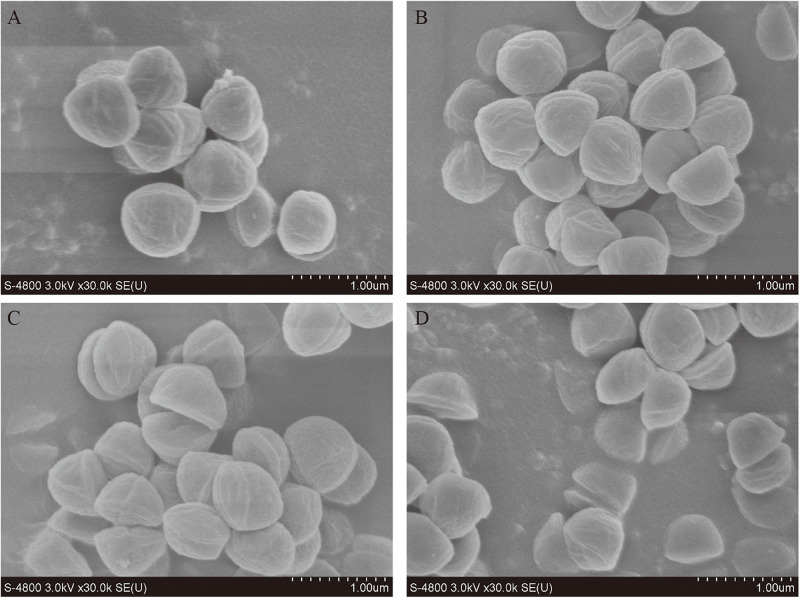
Scanning electron microscopy (SEM) observation of *S. xylosus* treated with nisin and PLA alone and in combination. **(A)** Control cells treated with 0.9% NaCl, **(B)** cells treated with nisin (50 IU/mL), **(C)** cells treated with PLA (5 mg/mL), and **(D)** cells treated with the combination of nisin (50 IU/mL) and PLA (5 mg/mL).

The morphological changes of *S. xylosus* were directly observed through SEM, to clarify the antibacterial effects of nisin and PLA alone or in combination (Figure 3). Evident changes in cell morphology were observed which suggesting a severe cell damage. The *S. xylosus* sample without nisin or PLA treatment maintained a regular spherical shape and displayed a relative flat and smooth surface (Figure 3A). Cells treated with nisin exhibited irregular shape and cell shrinkage (Figure 3B). Similar observations were earlier reported by Tong et al. (2010) for *Streptococcus sanguinis* and *Lactobacillus acidophilus* treated with nisin. Surprisingly, some hemispherical-shaped cells with wrinkled outer surface were observed in the PLA-treating sample which just as the dividing cell was split in half, indicating a distinct antibacterial mechanism with nisin (Figure 3C). For nisin and PLA in combination (Figure 3D), a more significant damage was evidenced from collapsed and split cell or even cell debris. These alterations of surface properties in *S. xylosus* revealed that the inhibition of bacterial growth could be due to the disruption in membrane permeability and blocked division of cells, which further confirmed that the combination of nisin and PLA displayed strong antibacterial activity.

### Construction of Nisin and PLA Co-producing *L. lactis* Strain

**FIGURE 4:**
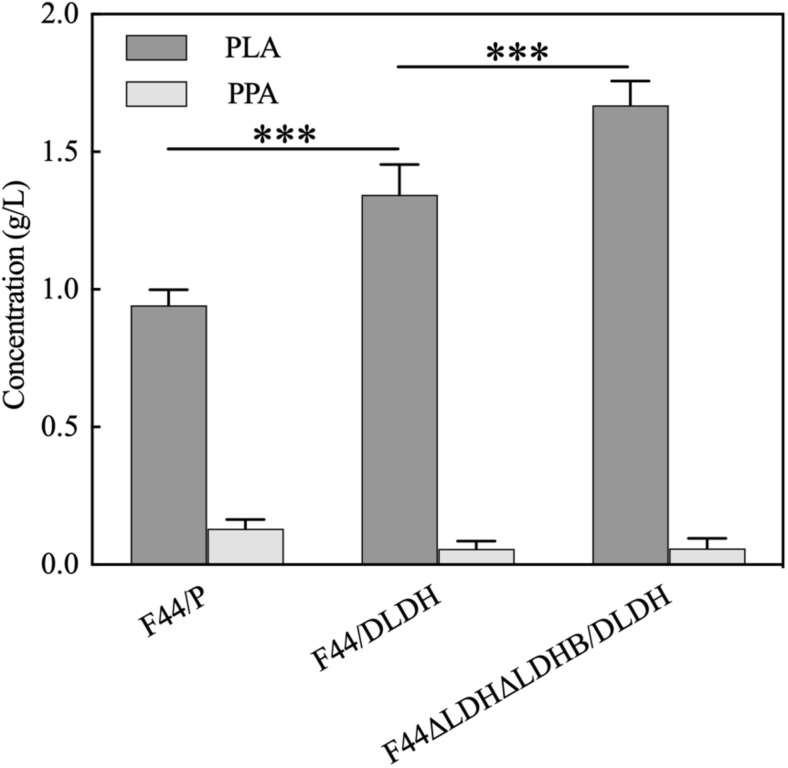
PLA and PPA concentrations of 12 h-culture supernatants of *L. lactis* F44/P and PLA-producing strain F44/DLDH, F44ΔLDHΔLDHB/DLDH. The error bar presented SD indicating the repeatability of the data in three-testing. The data were analyzed by One-way ANOVA. The yield of PLA in strain F44ΔLDHΔLDHB/DLDH was significantly different (***P < 0.01) from that in F44/P and F44/DLDH, respectively.

As the combination of nisin and PLA has shown strong antimicrobial activity and exhibited good performance in food preservation, we considered to construct a nisin-PLA co-producing strain, based on the nisin-producing strain *L. lactis* F44. Although F44 had the ability to produce PLA, the yield was only 54 mg/L in our previous work. Then 3 g/L PPA was added into the medium for PLA production. Additionally, a D-LDH mutant (D-LDH^*Y*52A^) (Ishikura et al., 2005), which exhibited higher catalytic activity and PPA preference, was also introduced into F44. As shown in Figure 4, the PLA yield of F44/P was 0.943g/L, which was dramatically improved compared with that of no PPA added. After the introduction of D-LDH^*Y*52A^, the PLA yield of F44/DLDH reached 1.344 g/L which further increased by 42.5% (*p* < 0.01), indicating a key role of D-LDH^*Y*52A^. The exogenous PPA was almost used up in the 12 h-fermentation supernatant.

It was considered that LDH in F44 is the L-type, which led to a mixture of L-PLA and D-PLA in the production of F44/DLDH. It motivated us to knock out the two main lactate dehydrogenases LDH and LDHB in F44. The LDH-deficient strain F44ΔLDHΔLDHB was constructed *via* a selection/counter-selection tool based on 5-FOA sensitivity (Solem et al., 2008). And then, a recombinant strain F44ΔLDHΔLDHB/DLDH (Supplementary Figure S1) was obtained by overexpression of D-LDH^*Y*52A^. An enhanced yield of PLA (1.670 g/L) was detected which had increases of 24.3% (*p* < 0.01) and 77.1% (*p* < 0.01) than F44/DLDH and the control F44/P, respectively. Notably, the recombinant strain F44ΔLDHΔLDHB/DLDH had a considerable production of PLA, and we further explored its antibacterial activity.

### Determination of the Bacteriostatic Efficacy of Fermentation Supernatants

**FIGURE 5:**
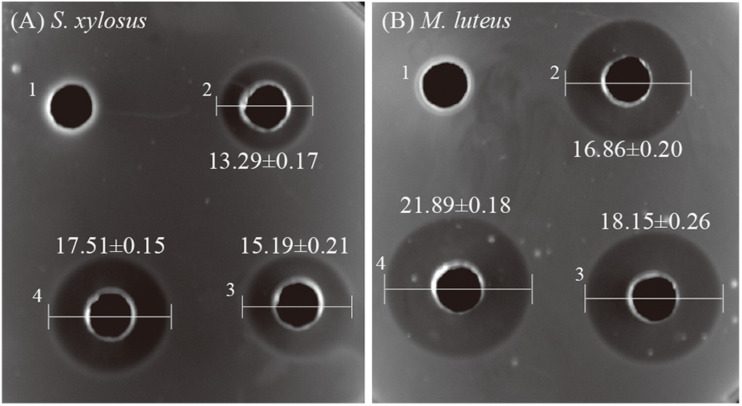
Results of agar-diffusion experiment with the fermentation broths of nisin and PLA co-producing *L. lactis* strains. *S. xylosus* **(A)** and *M. luteus* **(B)** were served as the indicators. The wells (6 mm) of the plates were added with 80 μL of (1) normal saline; (2) *L. lactis* F44/P; (3) *L. lactis* F44/DLDH; and (4) *L. lactis* F44ΔLDHΔLDHB/DLDH. Three repeated measurements were conducted. The differences between the four groups were compared by One-way ANOVA. The diameter of inhibition zone of strain F44ΔLDHΔLDHB/DLDH was significantly different (*P* < 0.01) from that in F44/P, F44/DLDH and control, respectively.

The antibacterial activities of fermentation supernatants of nisin and PLA co-producing strains were shown in Figure 5. The inhibitory activities of the constructed strains against *S. xylosus* and *M. luteus* were as follows: F44ΔLDHΔLDHB/DLDH > F44/DLDH > F44/P. After the introduction of D-LDH^*Y*52A^, the diameters of inhibition zones of F44/DLDH were increased 14.3% (*p* < 0.01) and 7.7% (*p* < 0.01) against *S. xylosus* and *M. Luteus*, respectively, when compared to F44/P. The inhibitory effects were further raised [31.8% (*p* < 0.01) and 29.8% (*p* < 0.01) increases against *S. xylosus* and *M. luteus*, respectively] for F44ΔLDHΔLDHB/DLDH. The antibacterial effect was in accordance with the findings of PLA contents of the recombinant strains, revealing a valuable fermentation product of the constructed strains.

## Discussion

Nisin, produced by certain *Lactococcus lactis* ssp. *lactis*, is the only bacteriocin allowed to be used as food additive which is generally recognized as safe (GRAS) by FDA. And it has strong inhibitory effect on many Gram-positive bacteria, i.e., food spoilage bacteria and pathogens (Turner et al., 2004; Nguyen and Mittal, 2007; Costas Malvido et al., 2016), contributing to the wide application in food industry as preservative. While nisin exhibited little effect on Gram-negative bacteria or molds. Recently, growing evidence suggested PLA possessed a broad antimicrobial spectrum of activity against both Gram-positive and Gram-negative bacteria (Liu et al., 2020a; Zhou et al., 2020), as well as fungi (Lavermicocca et al., 2003a), especially the D-PLA (Xu et al., 2016), which showed potentials as natural food antiseptic agent (Mu et al., 2012). Moreover, PLA could be cost-effectively produced *via* lactic acid bacteria fermentation.

This research demonstrated the combination of nisin and PLA could provide better antibacterial effect against *M. luteus* and *S. xylosus* (Figure 1). Studies suggested that nisin show additive or synergistic effects when used in combination with other antimicrobial agents, such as lysozyme (Chung and Hancock, 2000), lactates (Nykanen et al., 2000), and essential oils (Razavi Rohani et al., 2011), which is consistent with our result. *S. xylosus*, is not only common in fermented meat and milk, but is also one of the main pathogen in clinical infection with strong drug resistance (Martuscelli et al., 2000). This finding may imply their potential applications in food preservation and clinical treatment of bacterial infection. Several studies displayed the mechanism of antibacterial activity of nisin (Tong et al., 2010; Barbosa et al., 2013). Nisin can interact with the cytoplasmic membrane to form transient pores, which allow the efflux of intracellular substances with low molecular weights, such as adenosine triphosphate, amino acids and ions. While the anti-bacterial mechanism of PLA has not been well characterized, although it was suggested that PLA could interrupt the membrane permeability and damage genomic DNA as an intercalator, when investigated in *Listeria monocytogenes* and *E. coli* (Ning et al., 2017). Interestingly, our results first demonstrated a different mode, PLA affected the division of *S. xylosus* which caused the dividing cell to split from the septum (Figure 3C), implying divisome as a target. we hypothesized that it was the shape might result in the difference, rod-shaped bacteria and the spherical cocci ones displayed distinct elongation and septation models (Pérez-Núñez et al., 2011), which remained to be investigated. Moreover, in the presented study, it is revealed that nisin and PLA target cell membrane and septum, respectively, indicating a possible synergistic antibacterial efficacy. Generally, the use of nisin and PLA in combination exhibit better antibacterial effects, indicating a positive action. It is a novel combination worth to investigate the potent of practical application as preservative.

Our previous study showed that nisin in cooperation with γ-aminobutytic acid could prolonged the storage of meat and strawberry (Liu et al., 2020b). Combined use of nisin and leucocin C could work effectively against *Listeria monocytogenes* in pasteurized milk (Fu et al., 2018). In this study, the further research on practical application of a combination of nisin and PLA was carried out. For the pork preservation assay, there was no significant difference in the microbial acounts between nisin alone and nisin-PLA in combination until the day 3(Figure 2A), which might be due to the insufficient preservation time (Liu et al., 2020b). We conjectured the combination of nisin and PLA might be more suitable to the long-term preservation of meat, which remained to be discussed further. What was different from the above is that nisin in combination with PLA performed greater anti-microbial activity in the storage of pasteurized milk inoculated with *S. xylosus* and strawberry, compared with nisin and PLA alone (Figures 2B,C). It was the result that PLA presented better inhibitory activity against *S. xylosus* than nisin contributing for pasteurized milk storage. Additionally, PLA can also inhibit the growth of mold to some extent (Lavermicocca et al., 2003a), which was the key factor resulting in the rot of strawberry. Although some literatures on the PLA production and function have been reported (Nykanen et al., 2000; Zhang and Li, 2018), few presented the combined use of nisin and PLA. Our results promised a combinational effect of nisin and PLA on the antimicrobial activity during food preservation. The combination of nisin and PLA exhibited a potential candidate as food preservative, as well as an expanded scope of application.

As the limitation of the low cost-efficient, no pure nisin was commercially available. Nisaplin, the first commercial preparation, contains only 2.5% (w/w) nisin which is usually the fermentation product. In more cases, the fermentation broth of nisin is immediately applied for its “food-grade quality” producer (Barbosa et al., 2017), which to a certain extent lowers the cost. The food-grade strain *L. lactis* F44, because of its nisin production capacity, was used to construct the nisin-PLA co-expression strain, and a new antimicrobial agent PLA was induced into this fermented broth. The results of PLA content determination and antibacterial assay verified that the recombinant strains can successfully produce PLA (Figure 4) and the fermented broth with higher PLA content presented superior inhibitory effects against *S. xylosus* and *M. luteus* (Figure 5). It demonstrated that the F44ΔLDHΔLDHB/DLDH was more suitable for commercial application in industrial fermentation.

The biosynthetic pathway of PLA is initiated from phenylalanine which was converted to PPA by transaminase and then was further reduced into PLA by LDH in LAB (Lavermicocca et al., 2003a). Our results showed that the addition of PPA in the culture significantly increased the yield of PLA (0.054 g/L for no addition and 0.943 g/L for 3 g/L PPA) in F44/P, implying a serious shortage of biosynthesis of PPA in F44, which is in accordance with the previous literature of Zhang et al. (Zhang et al., 2014a). In addition, LDH is also a key for the improvement of PLA production and various activity gives rise to the disparity in PLA production (Li et al., 2008). Interestingly, D-PLA has a better performance against microbials than L-PLA (Xu et al., 2016). These findings revealed that the introduction of D-LDH^*Y*52*A*^ in F44 significantly improved the conversion rate of PPA to PLA (an increase of 42.5%). However, only L-LDH exists in F44, which leads to a mixture of L-PLA and D-PLA in the fermentation broth of F44/DLDH. Then, we deleted the two main LDH (LDH and LDHB) in F44 and induced D-LDH^*Y*52*A*^, where L-PLA could be reduced to a considerable extent, and a further 24.3% increase of PLA yield was obtained for F44ΔLDHΔLDHB/DLDH. Notably this study was a first preliminary attempt to endow a nisin-producing *L. lactis* strain with capacity of PLA synthesis using PPA as substrate. Since the synthesis of nisin is not involved in central carbon metabolism in *L. lactis*, further studies on realizing *de novo* synthesis of PLA from glucose was recommended to make co-production of nisin and PLA more cost effective.

## Conclusion

This study found that the cooperation of nisin and PLA performed a significant antibacterial activity and exhibited high potential as food preservative. Moreover, it is the first study to construct the nisin and PLA co-expressing engineered *L. lactis* and the fermentation product was demonstrated to be effective against bacteria. In contrast with nisin, PLA showed a different mode of action, contributing to the combinational antibacterial activity of nisin and PLA. The present study describes the potent application of the engineered *L. lactis* co-produing nisin and PLA, and gives insight to the development of natural food preservatives.

## Data Availability Statement

The original contributions presented in the study are included in the article/Supplementary Material, further inquiries can be directed to the corresponding author/s.

## Author Contributions

JQ and JL designed the experiment and guided the writing. QS and RH performed the major experiments and wrote the manuscript. HX, JM, and RX provided the additional experimental assistance and helped to revise the manuscript. All authors contributed to the article and approved the submitted version.

## Conflict of Interest

The authors declare that the research was conducted in the absence of any commercial or financial relationships that could be construed as a potential conflict of interest.
