# Supplementary material for: Combinational Antibacterial Activity of Nisin and 3-Phenyllactic Acid and Their Co-production by Engineered Lactococcus lactis
Source: Front Bioeng Biotechnol. 2021 Feb 5;9:612105. doi: 10.3389/fbioe.2021.612105 (PMC7901885; doi:10.3389/fbioe.2021.612105)
Supplement: Supplementary file 1 [file Data_Sheet_1.docx]

Supplementary Material

# Supplementary Figures and Tables

## Supplementary Figures


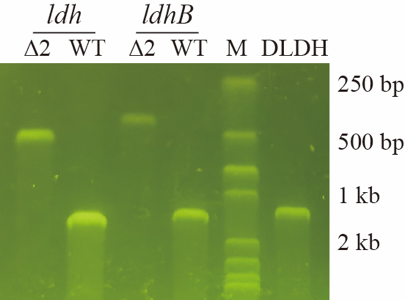


**Supplementary Figure S1.** Genomic DNAs from F44 (WT) and the recombinant strain F44ΔLDHΔLDHB/DLDH (Δ2) were used as templates for PCR reactions. The oligonucleotide primers used for gene knockout verification are located in the regions flanking the target gene. If the gene is present in the genome, a ~1.4 kb product should be amplified. While a smaller product can be obtained if the gene is deleted. The primers used to verify the overexpression of DLDH are located in the plasmid, amplifying a ~1.2 kb product.

## Supplementary Tables

**Supplementary Table S1.** Strains and plasmids used in this study.

| **Strain/Plasmid** | **Relevant properties** | **Reference** |
| --- | --- | --- |
| **Strains** |  |  |
| *L. lactis* F44 | Nisin Z producer, derived from *L. lactis* YF11 | (Zhang et al., 2014) |
| F44/P | *L. lactis* F44 with pLEB124 | This study |
| F44/DLDH | *L. lactis* F44 carrying pLEB124-DLDH | This study |
| F44ΔLDHΔLDHB | *L. lactis* F44 with the deletion of *ldh* and *ldhB* | This study |
| F44ΔLDHΔLDHB/DLDH | Strain F44ΔLDHΔLDHB carrying pLEB124-DLDH | This study |
| *E. coli* TG1 | Used for vector construction | This study |
| *M. luteus* ATC10240 | Indicator strain for the anti-bacterial activity | This study |
| *S. xylosus* | Indicator strain for the anti-bacterial activity | This study |
| **Plasmids** |  |  |
| pLEB124 | *L. lactis* secretion vector harboring promoter P45, erythromycin resistant | This study |
| pLEB124-DLDH | *d-ldh^Y52A^* cloned into pLEB124 | This study |
| pCS1966 | Vector for gene knockout in *L. lactis* | (Solem et al., 2008) |
| pCS1966-LDH | Upstream and downstream sequences of *ldh* cloned into pCS1966 | This study |
| pCS1966-LDHB | Upstream and downstream sequences of *ldhB* cloned into pCS1966 | This study |

**Supplementary Table S2.** Primers used in this study.

| **Primers** | **Sequence (5′ –3′)** |
| --- | --- |
| d-ldh-F | AAAAAAATTAACTTAAGTAAGCTTATGAAAATTATTGCCTATGCT |
| d-ldh-R | AACCCGGGCCCTATATATGGATCCTTAGTCAAACTTAACTTGTGTGTC |
| 124-F | AGGGAACCTAGAATAGTGAA |
| 124-R | TTCATTCTGCTAACCAGTAAGGC |
| 124-UF | AGGGAACCTAGAATAGTGAA |
| 124-UR | TTCATTCTGCTAACCAGTAAGGC |
| ldh-up-F | TCCTGCAGCCCGGGGGATCCTTCGGACTTTCACAACCTG |
| ldh-up-R | GATTTTCTTTAATTCCTTTCAAA |
| ldh-down-F | GAAAGGAATTAAAGAAAATCAAATAAAAAGAGTTGGTTGAGATT |
| ldh-down-R | TTGTTGGCGGCCGCTCTAGATTTACAAGTATTGGTGGTGTCTT |
| ldh-yz-F | GCTATCGACAGGCCCTATT |
| ldh-yz-R | CCTAAGGCTGATGGTGTTAAC |
| ldhB-up-F | CGGGATCCAATTAAGAAACTCGAAGAAGAATT |
| ldhB-up-R | GGTTTGGATAGATTTTAGCTCCTTTTCAATTTATAT |
| ldhB-down-F | AAGGAGCTAAAA TCTATCCAAACCTTACAAACATAA |
| ldhB-down-R | GCTCTAGATTTTAGGGGCCACAGTCA |
| ldhB-yz-F | AGGAGGAGATAGAATTGGCT |
| ldhB-yz-R | ATGGTTTATCTACTTTAGTCTTTCA |
| 1966-F | CTCACTCATTAGGCACCCC |
| 1966-R | GAGATAACTGATGAACTATGGGAC |
| orop-em-F | AAAGCCATGCGTCTGA |
| orop-em-R | TTTGGTGCGTTTCTGC |

**Reference**

Solem, C., Defoor, E., Jensen, P.R., and Martinussen, J. (2008). Plasmid pCS1966, a new selection/counterselection tool for lactic acid bacterium strain construction based on the oroP gene, encoding an orotate transporter from *Lactococcus lactis*. *Appl Environ Microbiol* 74(15)**,** 4772-4775. doi: 10.1128/AEM.00134-08.

Zhang, Y.F., Liu, S.Y., Du, Y.H., Feng, W.J., Liu, J.H., and Qiao, J.J. (2014). Genome shuffling of *Lactococcus lactis* subspecies *lactis* YF11 for improving nisin Z production and comparative analysis. *J Dairy Sci* 97(5)**,** 2528-2541. doi: 10.3168/jds.2013-7238.
